# Complete Genome Sequences of Walnut-Associated *Xanthomonas euroxanthea* Strains CPBF 367 and CPBF 426 Obtained by Illumina/Nanopore Hybrid Assembly

**DOI:** 10.1128/MRA.00902-20

**Published:** 2020-11-05

**Authors:** Miguel Teixeira, Leonor Martins, Camila Fernandes, Cátia Chaves, Joana Pinto, Fernando Tavares, Nuno A. Fonseca

**Affiliations:** a CIBIO-Centro de Investigação em Biodiversidade e Recursos Genéticos, InBIO-Laboratório Associado, Universidade do Porto, Vairão, Portugal; b FCUP-Faculdade de Ciências, Departamento de Biologia, Universidade do Porto, Porto, Portugal; c INIAV, Instituto Nacional de Investigação Agrária e Veterinária, Unidade Estratégica de Investigação e Serviços de Sistemas Agrários e Florestais e Sanidade Vegetal, Oeiras, Portugal; University of Arizona

## Abstract

We present the complete genome sequences of two Xanthomonas euroxanthea strains isolated from buds of a walnut tree. The whole-genome sequences of strains CPBF 367 and CPBF 426 consist of two circular chromosomes of 4,923,218 bp and 4,883,254 bp and two putative plasmids of 45,241 bp and 17,394 bp, respectively. These data may contribute to the understanding of *Xanthomonas* species-specific adaptations to walnut.

## ANNOUNCEMENT

*Xanthomonas* is a genetically diverse genus of bacteria comprising etiological agents of several plant diseases affecting crops worldwide and causing large yield losses and negative economic impacts (1–5). Xanthomonas arboricola is a species known to cause pathologies in several fruit trees, including walnut trees (Juglans regia L.). The occurrence of multiple *Xanthomonas* lineages within the same walnut host has revealed a consortium of walnut-associated *Xanthomonas* bacteria distinct from Xanthomonas arboricola pv. juglandis (5). More recently, based on phenotypic and genomic analyses, such as average nucleotide identity (ANI) and digital DNA-DNA hybridization (dDDH), it has been proposed that some of these bacteria are members of a new species designated Xanthomonas euroxanthea, which includes both pathogenic and nonpathogenic strains on walnut (6). Here, we sequenced the whole genome of two strains belonging to this new *Xanthomonas* species, CPBF 367 (LMG 31036 = CCOS 1890) and CPBF 426 (LMG 31038 = CCOS1892). These genomes should contribute to unveiling the ecology, evolution, and virulence of strains belonging to this new *Xanthomonas* species and disclose niche-specific adaptations.

Both strains were isolated in April 2016 in Loures, Portugal, from asymptomatic dormant buds of a walnut tree known to develop symptoms of walnut bacterial blight during the growing season. Characteristic mucoid yellow colonies were grown on yeast dextrose carbonate (YDC) agar medium, and pure colonies were further cultured in liquid medium from which an aliquot was cryopreserved at −80° in YDC with 30% glycerol as previously detailed (7).

DNA extraction for sequencing was carried out from cryopreserved bacteria recovered on bacterial culture medium M2 (yeast extract, 2 g liter^−1^; Bacto peptone, 5 g liter^−1^; NaCl, 5 g liter^−1^; KH_2_PO_4_, 0.45 g liter^−1^; Na_2_HPO_4_ 12H_2_O, 2.39 g liter^−1^) at 28°C and 100 rpm for 48 h. DNA was extracted using the E.Z.N.A. bacterial DNA purification kit (Omega Bio-tek, Norcross, GA) and sequenced with Illumina and Oxford Nanopore Technologies (ONT) MinION platforms. Illumina sequencing was outsourced to GATC Biotech, AG (Konstanz, Germany), using an Illumina HiSeq instrument with a standard 2 × 150-bp paired-end library protocol. This resulted in 8,413,466 and 6,494,807 reads, with 506× and 394× coverage for CPBF 367 and CPBF 426, respectively. For Nanopore sequencing, libraries were prepared with the SQK-LSK109 kit and multiplexed using the EXP-NBD104 barcoding kit. Sequencing was performed on a MinION sequencer using an R9.4.1 flow cell. Reads were base called and demultiplexed using Guppy v3.4.1 (high accuracy base-calling mode) and produced for CPBF 367 (18,857 reads with a mean length of 6,386 nucleotides [nt] [±8,299] and a maximum read length of 92,631 nt) and CBPF 426 (19,997 reads with a mean length of 5,895 nt [±6,300] and a maximum read length of 74,948 nt). The reads were assembled *de novo* following a hybrid Nanopore-Illumina approach using Unicycler v0.4.8 (8) and annotated with PGAP v2020-03-30.build4489 (9). Assembly metrics were calculated using QUAST v5.0.2 (10). The assembly quality and completeness were assessed using BUSCO v4.0.6 (parameters: lineage xanthomonadales_odb10) (11), which indicated that the CPBF 367 and CPBF 426 genomes are both >99.8% complete. Default parameters were used for all software unless otherwise noted.

The assembly of CPBF 367 has a total length of 4,968 Mb, comprising 2 circular contigs of 4,923,218 and 45,241 bp with a G+C content of 65.81%. The annotation indicates 4,014 coding genes in a total of 4,174 genes from 4,094 coding DNA sequences (CDSs), with 2 complete sets of rRNAs (5S, 16S, and 23S rRNAs), 56 tRNAs, 18 noncoding RNAs (ncRNAs), and 80 pseudogenes. For CPBF 426, the assembly has a total length of 4,900 Mb, comprising 2 circular contigs of 4,883,254 and 17,394 bp with a G+C content of 65.85%. The annotation revealed 4,010 coding genes in 4,157 genes from 4,080 CDSs, with 2 complete sets of rRNAs, 53 tRNAs, 18 ncRNAs, and 70 pseudogenes. Table 1 contains a summary of the two complete genome sequences.

**TABLE 1:** Summary of sequencing data and genome statistics

Characteristic	Data for strain:	
	CPBF 367	CPBF 426
ENA/GenBank accession number	GCA_903989455	GCA_903989465
No. of ONT reads	18,857	19,997
ONT read length (nt) (mean ± SD)	6,386 ± 8,299	5,895 ± 6,300
No. of Illumina reads	8,413,466	6,494,807
Illumina read length (nt)	2 × 150	2 × 150
Mean coverage (×)	532	415
G+C content (%)	65.81	65.85
*N*_50_ (bp)	4,923,218	4,883,254
No. of sequences	2	2
Genome size (bp)	4,968,459	4,900,648
Chromosome size (bp)	4,923,218	4,883,254
Plasmid size (bp)	45,241	17,394
No. of genes (total)	4,174	4,157
No. of CDSs (total)	4,094	4,080
No. of genes (coding)	4,014	4,010
No. of CDSs (with protein)	4,014	4,010
No. of genes (RNA)	80	77
No. of rRNAs (5S, 16S, 23S)	2, 2, 2	2, 2, 2
No. of complete rRNAs (5S, 16S, 23S)	2, 2, 2	2, 2, 2
No. of tRNAs	56	53
No. of ncRNAs	18	18
No. of pseudogenes	80	70
No. of CDSs (without protein)	80	70

OrthoANI v1.40 (12) indicates an average nucleotide identity value higher than 97%, assigning these strains to *X. euroxanthea*, together with a previously reported strain, CPBF 424 (13, 14). Interestingly, while *X. euroxanthea* CPBF 424 holds an apparently functional T3SS and was shown to be pathogenic in walnut plantlets, *X. euroxanthea* strains CPBF 367 and CPBF 426 were deficient for most T3SS operon genes, suggesting that these strains are nonpathogenic in walnut, which was confirmed in walnut pathogenicity assays for strain CPBF 367 (6). Altogether, the distinct genomic repertoires of these *X. euroxanthea* strains (CPBF 367 and CPBF 426), in comparison to those of X. arboricola pv. juglandis strains, will provide valuable genomic data for a better understanding of the pathogenicity and virulence of walnut-associated *Xanthomonas*.

### Data availability.

The raw data and assembled/annotated sequences have been deposited in the European Nucleotide Archive (ENA). The study accession number is PRJEB39139. Illumina reads, MinION reads, and the assembled genome are accessible under the accession numbers ERX2780809, ERX4296808, and GCA_903989455, respectively, for CPBF 367 and ERX2780811, ERX4296809, and GCA_903989465, respectively, for CPBF 426. Supplementary data are accessible via the BioStudies database with the accession number S-BSST433.
